# Indoor Air Quality Analysis Using Deep Learning with Sensor Data

**DOI:** 10.3390/s17112476

**Published:** 2017-10-28

**Authors:** Jaehyun Ahn, Dongil Shin, Kyuho Kim, Jihoon Yang

**Affiliations:** 1 Data Labs, Buzzni, Seoul 08788, Korea; jaehyunahn@sogang.ac.kr; 2 Department of Computer Science and Engineering, Sogang University, Seoul 04107, Korea; shindi91@sogang.ac.kr (D.S.), ekyuho@sogang.ac.kr (K.K.)

**Keywords:** deep learning, time series prediction, atmospheric observation system

## Abstract

Indoor air quality analysis is of interest to understand the abnormal atmospheric phenomena and external factors that affect air quality. By recording and analyzing quality measurements, we are able to observe patterns in the measurements and predict the air quality of near future. We designed a microchip made out of sensors that is capable of periodically recording measurements, and proposed a model that estimates atmospheric changes using deep learning. In addition, we developed an efficient algorithm to determine the optimal observation period for accurate air quality prediction. Experimental results with real-world data demonstrate the feasibility of our approach.

## 1. Introduction

With the proliferation of cheap but reasonably accurate sensors, indoor air quality can be determined by measuring various factors (e.g., fine dust density) through the sensors installed in a given space. Such measurements can be used to detect changes in the atmospheric state. Air quality can change sharply based on variables such as the entrance of people, the use of air conditioners and radiators, and the rate at which the air quality returns to its base state when the variable is removed. As such, a model designed to predict changes in indoor air quality must be able to take into account the various impacts of many variables. It also means that the model must be able to calculate the volume of the space in which it is being applied, as well as the thermal conductivity of other objects within the space, among other things. Furthermore, the model needs to calculate these values for any unspecified number of objects, which makes the development of such a model very difficult.

Due to the above difficulties, until recently, many indoor air quality control systems have controlled the variables by establishing thresholds. This method applies a given operation when current conditions exceed preset values, regardless of the number of variables or obstacles in the space. For example, in the case of a refrigerator, the air within the refrigerator is regulated via a cooling system that turns on when the temperature rises above a set value, and turns off when the temperature drops below a set value. Air quality is controlled in the same way in precision machines. In the case of anaerobic incubators used for microbiological experiments, if the oxygen concentration exceeds a preset critical point, the concentration of nitrogen gas and carbon dioxide gas is increased to maintain the anaerobic organism culture environment.

However, the use of critical points is not suitable for precision instruments which are highly affected by minute air quality changes. In many precision instruments, if the critical point is exceeded rapidly, the control is likely no longer meaningful. For example, in an incubator for biological experiments, microorganisms cannot survive after they have passed beyond the critical point of survival, even if the environment is restored to its prior state.

In addition, the critical point measurement method does not take into consideration the interaction between the surrounding environment and the given space. Generally, when the indoor measurement cycle is set at a given time interval, the variable with the greatest effect on the space is the sun. The phase changes of the sun affect the troposphere and as a result has a lasting impact on numerous measurable factors, including temperature, humidity, light quantity, and fine dust. This example implies that a single variable does not affect only one specific factor but instead creates a complex, powerful, and correlated variable. However, in the case of the critical point measurement method, it is difficult to grasp or distinguish the cause of the variable because it operates only for one specific factor. For example, when the amount of light exceeds a given critical point, the observer cannot distinguish whether this value is due to a person entering the space or the change in the sun’s positioning.

This paper recognizes the limitations in the conventional method described above and proposes a model for predicting the time series data using machine learning. In order to handle the time series data of measurements from diverse sensors, two deep learning methods are adopted long short-term memory (LSTM) [1] and gated recurrent units (GRU) [2]. (Detailed descriptions on the algorithms are given in the references.) All of the measurements are considered together in learning in a bid to exploit any relationships among them, and produce a model that correctly predicts the measurements (i.e., air quality) at the next time step.

The rest of the paper is organized as follows: Section 2 describes previous work related to this research. Section 3 gives detailed explanations on our method and the sensor data. Section 4 presents the results of the experiments that were designed to evaluate the performance of our method. Section 5 concludes with a summary and discussion of some directions for future research.

## 2. Related Work

### 2.1. Air Quality Prediction Using Machine Learning

Several attempts have been made to predict air quality using machine learning. The major method adopted is the regression model. In Allen et al.’s paper [3], indoor particle matters were measured and applied to regression models for their prediction in the future. Also, air quality studies on indoor nitrogen dioxide and exhaust gas concentrations have established regression methods to predict the ultrafine particles (PM_2.5_) index in a way that easily visualizes the trend line [4]. In addition, external seasonal conditions were combined with a regression model to predict the temperature inside a greenhouse [5].

### 2.2. Time Series Data Learning

The first use of machine learning to predict time series data was an attempt to recognize acoustic signals and the surrounding environment. In the classification of acoustic signals, speech recognition models were constructed using the hidden markov model (HMM), which is based on conditional probability and support vector machines (SVM) [6]. With the emergence of deep learning techniques, time series analysis methods based on circular (or recurrent) neural network structures have been developed.

In essence, the learning model of circular neural network structures utilizes temporal information in the hidden layer. As such, the circular neural network structure is able to classify and combine past and current information [7]. However, this circular structure has been found to be unsuitable for long-term memory dependency, due to the vanishing or exploding gradient problem. The long short-term memory (LSTM) network appears to solve these issues by controlling the output of hidden units using memory cells and gates [1]. Yet another popular circular neural network model for time series data is gated recurrent units (GRU) [2]. The GRU is a network model similar to LSTM, but it reduces the number of gates and effectively reduces the number of parameters. These models are trained by the backpropagation through time (BPTT) algorithm reflecting the time sequence. Both LSTM and GRU are currently the best circular network models, but there still exists some debate regarding the performance between them [8].

## 3. Indoor Air Quality Prediction System Using Deep Learning

We applied both LSTM and GRU models in our experiments. Here we describe the experimental setup (including the sensors), data refinement, and the methodology for establishing and modeling the comparative group and the control group of machine learning models.

### 3.1. Sensor Data

**Figure 1:**
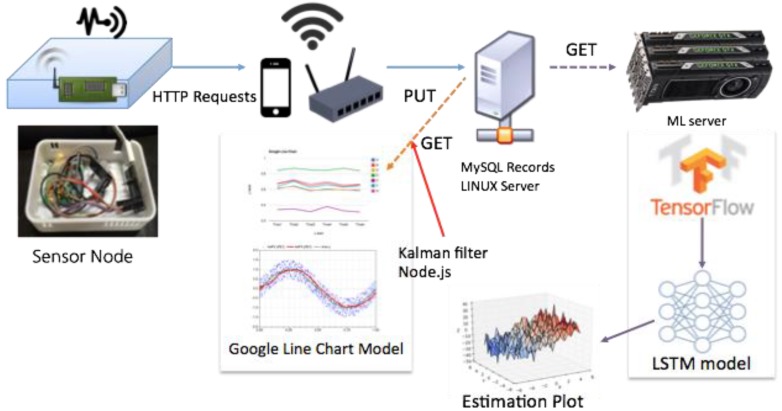
Module diagram for the periodic measurement and transfer of air quality data.

This experiment measures six atmospheric factors: carbon dioxide, fine dust, temperature, humidity, light quantity, and volatile organic compounds (VOC). The sensor measurement module periodically transmits the data collected to the server. The server transmission cycle is one minute. During one cycle, the module measures the air quality based on the six aforementioned factors and sends the data to the Linux server (Figure 1) [9].

#### 3.1.1. Sensor Instrument for Data Collection

**Figure 2:**
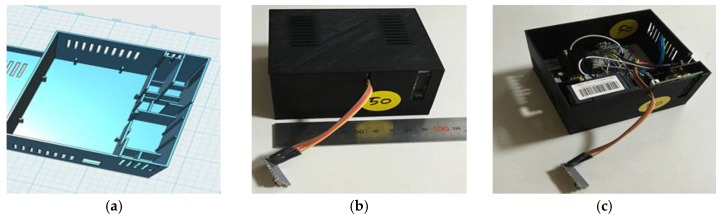
Sensor meter case made using 3D printer (**a**) and sensor meter (**b**,**c**).

Six types of sensor nodes are installed on the Arduino board for measuring indoor air quality. Figure 2 shows the sensor meter, and Table 1 provides details on each sensor node. (The effective concentration range of the fine dust detector is within 500 µg/m^3^.) The sensor nodes in Table 1 are configured as shown in Figure 1. The server stores the data, which is transmitted through each cycle of a minute, in the MySQL database.

**Figure 3:**
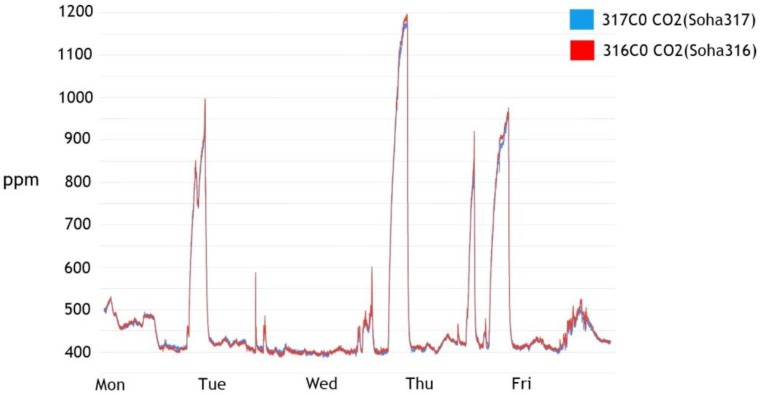
CO_2_ measurements of four sensor meters in Figure 3.

The validation of the sensors was confirmed by the concept of a “collaborative” sensor (a combination of several sensors). Figure 3 is the two sensor meters placed in one place whose CO_2_ measurements are shown. As we can see, all of the sensor values followed a similar trend with high measurement accuracy (the difference between the highest and lowest values is small and within the error range).

Though the six air quality factors were measured independently, they might work together. Naturally, temperature and light density are related, which may or may not influence other factors as well. We thus try to predict the air quality accurately considering the potential relationships among various factors using machine learning.

**Figure 4:**
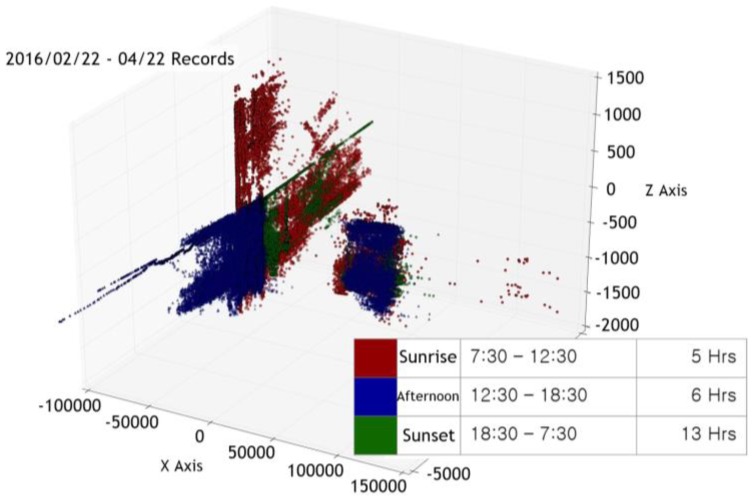
A visualization of six air quality indicators collected from 22 February to 22 April 2016 in 3D.

**Figure 5:**
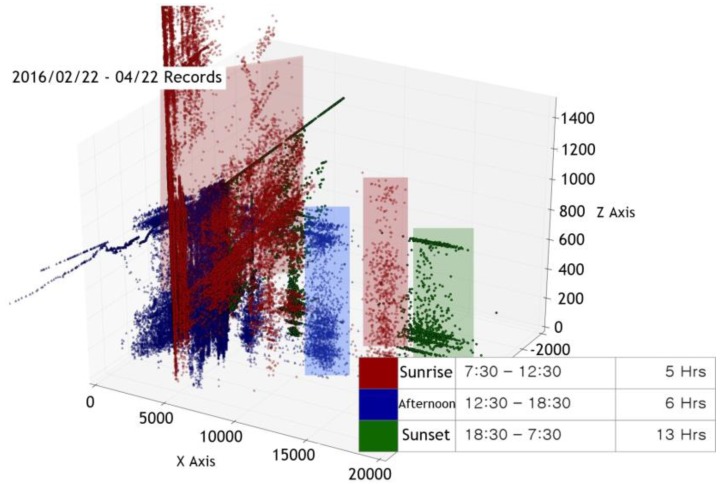
Part of Figure 4 showing well-clustered data points.

**Figure 6:**
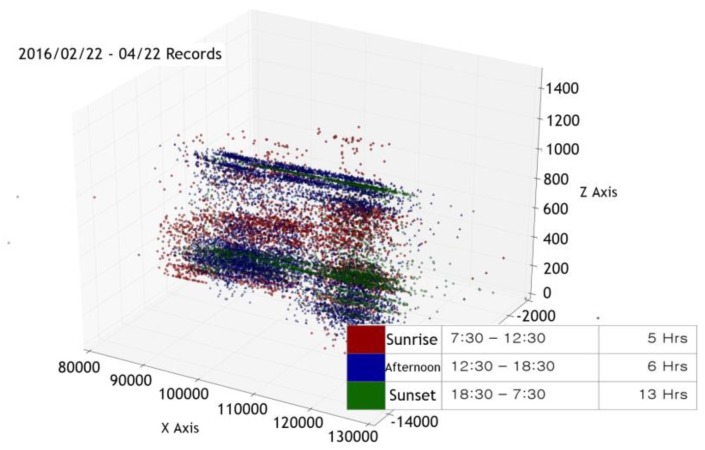
Part of Figure 4 showing interspersed data points.

We collected sensor measurement data over about seven months at three different times of the day: sunrise (7:30 a.m. to 12:30 p.m.), afternoon (12:30 p.m. to 6:30 p.m.), and sunset (6:30 p.m. to 7:30 a.m.). The data was visualized using principal components analysis (PCA) [10]. Figure 4 is the visualization of the six measurement data collected from 22 February 2016 to 22 April 2016, into a three-dimensional space defined by PCA. We can see that the points are distributed around the position of the sun on the whole. However, the data points in some regions are either well distributed by time (clustered by different colors) or not, as we can see in Figure 5 and Figure 6. We surmise that understanding this type of data requires more advanced machine learning methods than simple linear regression models.

#### 3.1.2. Data Preparation

As described previously, the sensor measurements were collected periodically via MySQL at six sensor nodes (Figure 1). Details of the data are summarized in Table 2.

**Figure 7:**
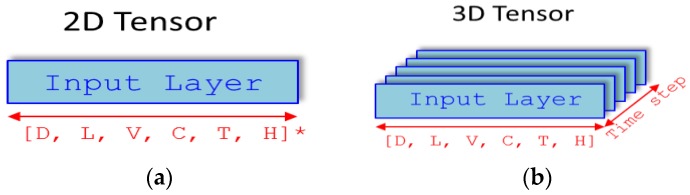
Two-dimensional (**a**) and three-dimensional tensor (**b**) representation of data.

For our experiments, a sensor measurement sample was created for each time step as a two-dimensional tensor by merging the values from the sensor nodes, and arranged as a set of six air quality values. The resulting sample set is a three-dimensional tensor, which is basically a bundle of two-dimensional tensors in time intervals, as shown in Figure 7. Given that the measurement period of the experiment is one minute, it has a volume of 1×timestep size. When the time step of this three-dimensional tensor is set as t, the time series prediction model attempts to predict the state of the vectors when t+1 time has elapsed.

The training and test data set of the three-dimensional tensor is determined using 10-fold cross- validation. A total of 21,781,467 CSV records with 10 measurements were tracked [11]. Among them, six measurements were taken for air quality: fine dust (D), light amount (L), volatile organic compound (VOC), carbon dioxide (CO_2_), temperature (T), and humidity (H). These measurements produced the training data of a [t×6×1]×299,596 three-dimensional tensor, and the test data of a [t×6×1]×33,289 three-dimensional tensor, given the time step parameter *t*. Algorithm 1 shows the algorithm (or process) for generating the three-dimensional tensor.

**Algorithm 1.** 3D tensor construction**Input**: CSV record ${\left\{v_{1,2,3,\ldots ,9,10}\right\}}_{record}$,    Fold rate r,    Time step t Initialize Model parameter fold rate is used for K-fold cross validation1.1. Select_fecature() ← Select ${\left\{v_{1,2,3,\ldots ,5,6}\right\}}_{record}$2. Set_feacutre_vector() ← Sort ${\left\{v_{1,2,3,\ldots ,5,6}\right\}}_{record}$ through time3. ${\left\{x_{v\times t}\right\}}_{record}$ ← Pile ${\left\{x_{v\times 1}\right\}}_{record}$ with t times in order4. ${\left\{x_{v\times t},y_{v\times 1}\right\}}_{test}/{\left\{x_{v\times t},y_{v\times 1}\right\}}_{train}$ ← Separate ${\left\{x_{v\times t}\right\}}_{record}$ by fold rate r **Output**: Training Dataset/Test Dataset, ${\left\{x_{v\times t},y_{v\times 1}\right\}}_{train}/{\left\{x_{v\times t},y_{v\times 1}\right\}}_{test}$

### 3.2. Machine Learning Models for Time Series Data

We briefly introduce three machine learning models for handling time series data such as our sensor data. One is a linear regression model and the other two are deep learning models, LSTM and GRU. (See the reference for detailed descriptions on the models).

#### 3.2.1. Linear Regression 

A linear regression model considers the linear relationship between data points and constructs a model that can describe or predict the value of a dependent variable using independent variables. Multiple regression analysis is a model construction method that confirms Equation (1):

(1) $$Y=\beta_{0}+\beta_{1}X+\beta_{2}X_{2}+\ldots +\beta_{k}X_{k}+\epsilon$$

Here, $X_{1},X_{2},\ldots ,\;X_{k}$ are independent variables, Y is a dependent variable, $\beta_{0},\beta_{1},\ldots ,\;\beta_{k}$ are unknown constants used as regression coefficients, and ϵ is the error term [12].

#### 3.2.2. Long Short-Term Memory Network

A long short-term memory network (LSTM network) applies the structure of a recurrent neural network (RNN). The LSTM structure has emerged to overcome the issue in which circular neural networks fail to store long-term historical information. The LSTM architecture regulates the storage of prior information using three gates: input, output, and forget gate [1]. The LSTM network model combines these three pieces of gate information to determine the amount of information to be stored from the past and to be transferred to the future.

As seen in Equation (2), the input gate controls the input data at the current time. In this case, $x_{i}^{t}$ is the input value received from the *i*th node at time t. $b_{h}^{t-1}$ denotes the result of the *h*th node at time t−1. $s_{c}^{t-1}$ denotes the cell state of the *c*th node at time t−1. ω is the weight, which is the weight value between the nodes. f is an activation function.

$$a_{l}^{t}=\overset{I}{\underset{i=1}{\sum }}\omega_{il}x_{i}^{t}+\overset{H}{\underset{h=1}{\sum }}\omega_{hl}b_{h}^{t-1}+\overset{C}{\underset{c=1}{\sum }}\omega_{cl}s_{c}^{t-1}$$

(2) $$b_{l}^{t}=f\left(a_{l}^{t}\right)$$

The output gate of Equation (3) is responsible for transferring the current value to the output node.

$$a_{\omega }^{t}=\overset{I}{\underset{i=1}{\sum }}\omega_{i\omega }x_{i}^{t}+\overset{H}{\underset{h=1}{\sum }}\omega_{h\omega }b_{h}^{t-1}+\overset{C}{\underset{c=1}{\sum }}\omega_{c\omega }s_{c}^{t-1}$$

(3) $$b_{\omega }^{t}=f\left(a_{\omega }^{t}\right)$$

Finally, in the forget gate, the current value is stored in the cell state as in Equation (4).

$$a_{\emptyset }^{t}=\overset{I}{\underset{i=1}{\sum }}\omega_{i\emptyset }x_{i}^{t}+\overset{H}{\underset{h=1}{\sum }}\omega_{h\emptyset }b_{h}^{t-1}+\overset{C}{\underset{c=1}{\sum }}\omega_{c\emptyset }s_{c}^{t-1}$$

(4) $$b_{\emptyset }^{t}=f\left(a_{\emptyset }^{t}\right)$$

The LSTM model was proposed to influence the current classification by the information in the time series data such as acoustic signals, and produced improved performance regarding the long-term memory dependency problem over standard RNNs.

#### 3.2.3. Gated Recurrent Unit Network

The gated recurrent unit (GRU) network is an LSTM variant with only two gates (reset and update) [2], implementing Equation (5).

$$z=\sigma \left(W_{z}x_{t}+U_{z}h_{t-1}+b_{z}\right)$$

$$r=\sigma \left(W_{r}x_{t}+U_{r}h_{t-1}+b_{z}\right)$$

$$m=\emptyset \left(W_{m}x_{t}+U_{m}(h_{t-1}\circ r\right)+b_{m})$$

(5) $$h_{t}=\left(1-z\right)h_{t-1}+z\circ m$$

In Equation (5), σ is a sigmoid function, $x_{t}$ is the input value at time t, $h_{t-1}$ is the output value at time t−1 and $W_{z},\;U_{z},\;W_{r},\;U_{r},\;W_{m},\;U_{m}$ are the weight matrices for each gate and cell memory. r represents the reset gate, which determines the rate at which the previous state is reflected in the input of the unit. z represents the update gate which holds the previous state of the output of the unit and adjusts information accordingly. ∘ represents element-wise products and ∅ is an activation function.

The GRU is simpler than LSTM, since it combines the forget and input gate of the LSTM network into a single update gate, and also combines the cell state and the hidden state into a single reset gate.

### 3.3. Indoor Air Quality Prediction Using GRU

As shown in Section 4, GRU networks produced the best performance. In the following section, we explain the details of our implementation of the GRU model.

#### 3.3.1. System Construction

In order to build a GRU learning model, it is necessary to determine various numerical parameters, including the number of hidden layers, the number of hidden layer nodes, the size of the time step, and the activation function. In this work, such parameters (except the time-step size, which was set by a search algorithm in Section 3.3.2 were chosen based on the results of 21 preliminary experiments. Two hidden layers of 1270 nodes with sigmoid activation function were used. The output layer computes the final output by applying the softmax function to the output of dense layer, wherein a two-dimensional representation is produced from the output of the upper hidden layer.

The adaptive moment estimation (ADAM) optimization algorithm [13] is employed in learning. ADAM is one of the most commonly used methods in deep learning algorithms. It adjusts the learning rate based on the mean and variance of the slope combined with the bias term. ADAM is a combination of the AdaGrad [14] and the RMSProp algorithms which work by increasing the learning rate that are not updated effectively over time.

**Figure 8:**
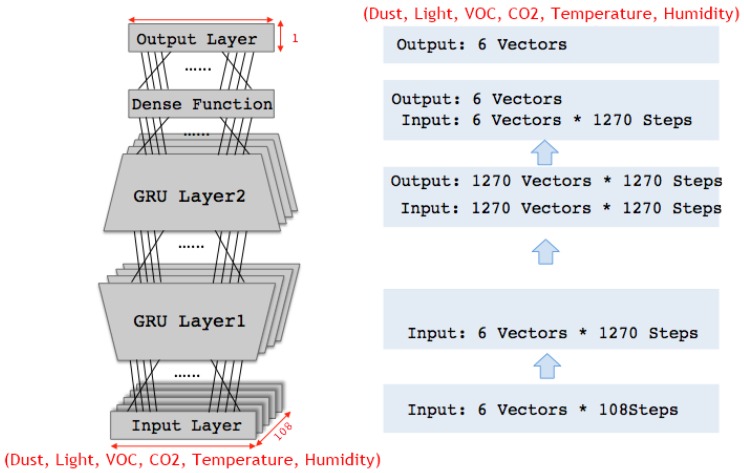
Gated recurrent units (GRU) network for air quality prediction.

The GRU network architecture and its learning algorithm are depicted in Figure 8 and Algorithm 2, respectively.

**Algorithm 2.** GRU Learning**Input**: Training Dataset ${\left\{x_{v\times t},y_{v\times 1}\right\}}_{train}$,    Input/Output values 6,    Hidden layer nodes 1270,    Time step t 1. GRU_model() ← Train model with ${\left\{x_{v\times t},y_{v\times 1}\right\}}_{train}$ and parameters (6, 1270, t)2. ${\left\{y_{v\times 1}\right\}}_{test}$ ← Predict output values based on GRU_model() with ${\left\{x_{v\times t},y_{v\times 1}\right\}}_{test}$ **Output**: Predicted values ${\left\{y_{v\times 1}\right\}}_{test}$ of test data

#### 3.3.2. Time Step Search 

Similar to other parameters, the performance of the model also depends on the size of the time step. Therefore, it is of importance to find the optimal time-step size. A brute force method would be constructing models with all possible time-step sizes and picking the best one. However, this entails an exorbitant overhead in learning.

Based on the training data and the machine learning model constructed by Algorithms 1 and 2, an effective algorithm for finding the best time step is designed as shown in Algorithm 3. Here, given a time-step size, the largest step size (within the limit of the given size) that yields the best prediction accuracy is obtained. Repeating this process, starting from the maximum step size, a series of time- step sizes with good performance is generated.

**Algorithm 3.** Time-step size search**Input**: Time-step size $t_{s}\left(t_{s}\geq 1\right)$ While ($t_{s}\geq 1$) {  Learn GRU_model() with $t_{s}$ by Algorithm 2  Get the accuracy k of the model on test data ${\left\{x_{v\times t},y_{v\times 1}\right\}}_{test}$  Record $t_{s}\;\&\;k$  Compute the maximum accuracy length
*l* within $t_{s}$ period  if ($l==t_{s}$)     $t_{s}=t_{s}-1$  else     $t_{s}=l$} **Output**: Time-step size candidates $t_{s_{1}},t_{s_{2}},\ldots ,t_{s_{t}}$

## 4. Experiments and Results

First, we compare the performance (in terms of prediction accuracy) of our model (GRU networks) with others (linear regression and LSTM networks). We also visualize the distributions of both actual and estimated data for easy interpretation of the results. Finally, we show the results of experiments for identifying the optimal time-step size of the GRU model, and compare with those of the brute force method.

### 4.1. Time Series Data Prediction 

Table 3 contains the performance of the GRU and LSTM models under different parameter settings. We counted a prediction as accurate if the difference between the actual and predicted values is less than 0.001. As we can see from Table 3, the GRU model outperformed the LSTM model. In particular, the best performance of the GRU model (shown in bold face, **84.69%**, experiment number 13) was significantly higher than that of the LSTM model (70.13%, experiment number 19). Though not shown in the table, we applied linear regression to the data and obtained 60.96% of accuracy. Therefore, the GRU model displayed a clear edge over the linear regression method as well. As far as the network architecture, the GRU model performed well with wide and shallow networks, without the overhead of deep architectures.

**Figure 9:**
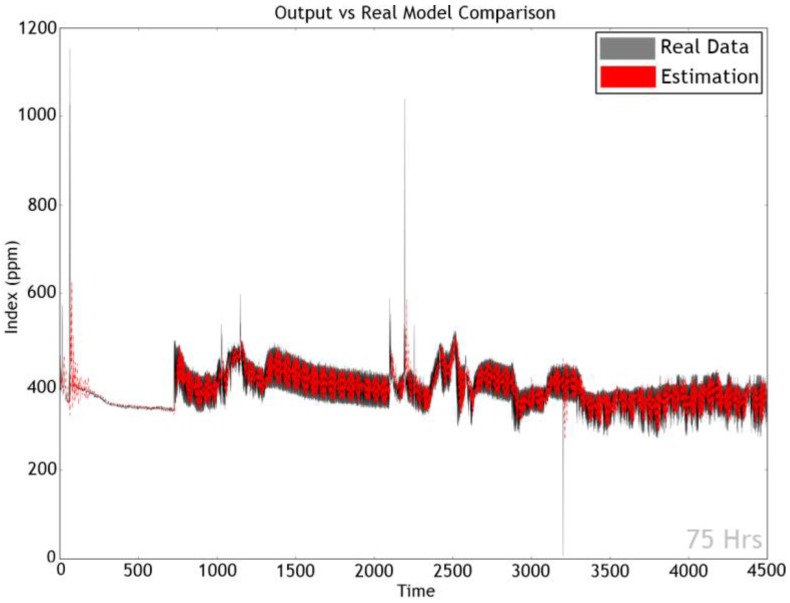
Analysis of dust data.

**Figure 10:**
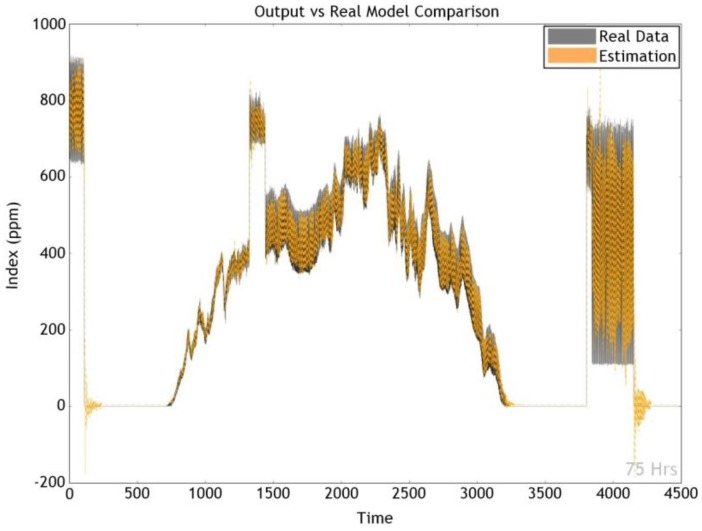
Analysis of CO_2_ data.

In order to verify the performance, the most accurate GRU model of experiment 13 was considered. Figure 9 and Figure 10 exhibit the actual as well as predicted values for dust and CO_2_ factors. As the model produced high accuracy of a 84.69%, the two graphs display very similar shapes and tendencies.

### 4.2. Optimal Timp Step Search 

First, Algorithm 3 was applied as described in Section 3.3.2 to a model with two hidden layers and 128 hidden nodes in a layer. The time-step sizes varied starting from 256 toward 1. The maximum performance difference was 7.9% depending on the time step-sizes selected.

**Figure 11:**
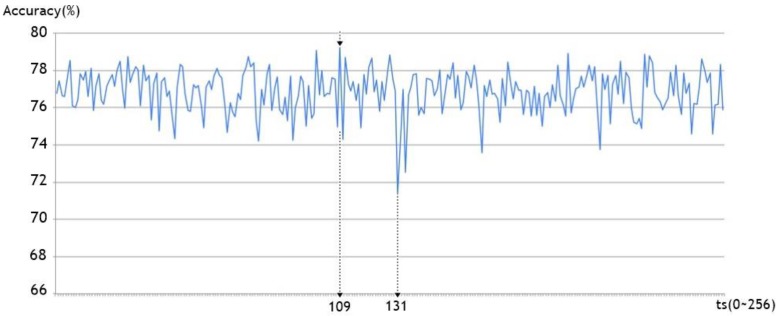
Performance of the GRU model with different time-step sizes.

For the brute force method, all of the time-step sizes between 1 and 256 were tried in learning (Figure 11). The time-step sizes with the best learning performance was 109 (learning performance of 79.25%), and the that with the lowest learning performance was 131 (learning performance of 71.34%).

Table 4 summarizes the performance of both approaches. As we can see, our method is significantly better than the brute force method in terms of the learning time and performance. In other words, it automatically finds the best time-step size quite efficiently, which results in an improved performance over the brute force method.

## 5. Conclusions

We proposed an air quality prediction system using sensor data and machine learning. We applied a composite model under the assumption that the data (i.e., diverse sensor measurements) interact with each other, and that the model presented in this paper is more efficient in prediction ability than the single linear regression method, and verified its performance. In addition, we proposed an algorithm that determines the optimal time-step size automatically for deep learning models. Our model demonstrated its feasibility and outstanding performance through experiments with a variety of parameter settings. We plan to incorporate additional sensor nodes and to apply more state-of-the-art machine learning algorithms.

## Figures and Tables

**Table 1:** Six sensor nodes used for air quality measurement.

Device Type	Model	Interface	Measuring Range
CO_2_ sensor	SH-300-DS	UART	0–3000/5000 ppm
Fine dust detector	PMS3003	UART	0.3–10 µm
Temperature/Humidity meter	SHT11	I2C	−40–125 °C/0–100%RH
Light sensor	GL5537	UART	5–200 kΩ (light resistance)
VOC sensor	MICS-VZ-89	UART	$H_{2}$ (100 ppm), I-butane (100 ppm)
CPU	ATMEGA328P	-	Connect to breadboard
Wi-Fi module	ESP8266	-	Connect to breadboard

**Table 2:** Summary of sensor data.

Collection Site	SK Corporation Jongro Building (Seoul, Korea)
Number of records	21,781,467
Size	1.36 GB (1,426,063 Bytes)
Collection period	60,504 h (22 February 2016~20 September 2016)
Value types	Six air quality variables (CO_2_, Dust, Temperature, Humidity, Light, VOC)

**Table 3:** Performance comparison between GRU and LSTM models.

Experiment Number	Learning Model	Basic Layers	Number of Basic Layers	Number of Hidden Nodes	Number of Hidden Layers	Total Number of Layers	Prediction Accuracy
1	GRU	in/out	2	128	1	3	79.26%
2	GRU	in/out	2	32	3	5	77.40%
3	GRU	in/out	2	32	2	4	67.55%
4	GRU	in/out	2	32	4	6	73.32%
5	GRU	in/out	2	32	4	6	72.13%
6	GRU	in/out	2	256	2	4	81.96%
7	GRU	in/out	2	256	1	3	81.34%
8	GRU	in/out	2	384	1	3	80.03%
9	GRU	in/out	2	16	4	6	70.39%
10	GRU	in/out	2	6	4	6	60.31%
11	GRU	in/out	2	384	3	5	81.58%
12	GRU	in/out	2	1536	3	5	83.16%
13	GRU	in/out	2	1270	2	4	84.69%
14	GRU	in/out	2	512	2	4	83.80%
15	GRU	in/out	2	1024	2	4	82.43%
16	GRU	in/out	2	1024	3	5	82.43%
17	LSTM	in/out	2	32	3	5	60.23%
18	LSTM	in/out	2	32	4	6	61.22%
19	LSTM	in/out	2	1024	3	5	70.13%

**Table 4:** Comparison between the time-step search algorithm and the brute force method.

	Optimal Time Step Search Algorithm	Brute Force Method
Number of time steps considered	134	256
Learning time	38 h	73 h
Maximum learning accuracy	79.22 (with size 100)	79.25 (with size 109)
Average earning accuracy	77.62%	76.85%
Time efficiency relative to the brute force method	1.92 times	1 times
